# Manual of Rational Pathology

**Published:** 1847-10

**Authors:** 


BRITISH AND FOREIGN

### MEDICAL REVIEW,

FOR OCTOBER, 1847.

### PART FIRST.

### Analytical and Critical Reviews.

### Art. I.

*Handbuch der rationellen Pathologie.* Von Dr. J. Henle, Professor der Anatomie und Physiologie in Heidelberg. Erster Band. Einleitung und allgemeiner Theil. Zweite unveränderte Auflage.—*Braunschweig,* 1846.

*Manual of Rational Pathology.* By Dr. J. Henle, Professor of Anatomy and Physiology in Heidelberg. First Volume. Introduction and General Part. Second and unchanged edition,—*Brunswick,* 1846. 8vo, pp. 357.

Professor Henle is well known as one of the most distinguished of the distinguished anatomists and physiologists of Germany. As a pathologist, also, he was introduced to our readers some years ago. Still continuing his attention to pathology he has, in conjunction with his colleague Professor Pfeufer, for some time past conducted a journal (Zeitschrift für rationelle Medicin) for the reception of such researches and observations as turn to account the recent progress of pathological anatomy, organic chemistry, physiology, and general anatomy in elucidating the nature of disease and the action of remedies; and now we have from his pen the work before us.

This work, the author tells us in his preface, is an attempt to arrange, in that form which the systematic genius of the Germans requires, the physiological facts which observation of the diseased body has brought to light, together with the theories and hypotheses to which they have given rise, in order to assign to them their place in the history of the development of science.

The fact that, soon after the appearance of this first volume of the work, a second edition was required, affords evidence of the high estimation in which the author's labours are held by his professional countrymen. Participating in this estimation and thinking the work calculated to clear the medical mind of a great deal of misconception and prejudice, we bring it under the notice of our readers by means of a pretty full abstract.

In the introduction the first subjects discussed are the *Methods in Medicine.* Two methods strive for the preference, viz. the *empirical* and the *rational.*

The first method describes the symptoms merely, without inquiring into their cause and intimate connexion ; its names are not definitions, but only proper names, and when it uses a word, as, for example, inflammation, it means by it nothing more than the combination of the symptoms of redness, heat, swelling, and pain. The adherents of this method trouble themselves as little with the mode of action of the causes and the remedies as with the conditions on which the symptoms depend. As to remedies they use those which have most frequently been followed by good effects. As in this case the certainty of the methods of cure is not determined by internal arguments, but only by the number of observations the practitioner has made, everything depends on collecting and making use of as extensive a series of observations as possible. Clinical experience is here the rule of treatment. Such is the *empirical method.*

The second method—the *theoretical, physiological,* or *rational*—considers the symptoms in their dependence on each other, and in their connexion with internal changes ; and these changes it views as the effects of external influences on the organic structure endowed with peculiar powers. In defining a disease, instead of the symptoms, it describes the state which it believes it has ascertained to be that on which the symptoms depend. It employs names which express the nature of the morbid changes, preferring, for example, such names as "increased plasticity," "hyperæmia," "stasis," &c., for inflammation, according to the prevailing opinions regarding the essential nature of that morbid process. As to remedies, theoretical medicine investigates their absolute powers and properties and their so-called physiological action, i. e. the mode in which they affect the substance and forces of the organism.

The diagnosis in empirical medicine, like that in the descriptive natural sciences, teaches nothing more than that the particular case, from its external marks, comes under this or that class of known cases ; the diagnosis in theoretical medicine is the synoptical and independent history of the particular case. To the empirical physician a definite combination of symptoms, appreciable by the senses, immediately indicates a *specific* mode of treatment ; to the theoretical physician an internal lesion, which is supposed to exist, first indicates the internal change to be effected, and thus, indirectly, the treatment by which it is supposed the required change may be effected.

If it were a question of choice between the two methods, it must be confessed that the empirical in principle promises most certainty ; for in a simple comparison of phenomena—a mere enumeration of *pros* and *cons*—there is less liability to error than in a process of reasoning, in which conclusion is founded on conclusion, and in which a defective link may vitiate the whole chain. Such a fact as that bark cures ague, on the contrary, is in itself so certain that it is quite indifferent how the nature of ague and the mode of action of bark are explained. But such indisputably certain remedies are rare. After an experience of 2000 years, doubts are still expressed as to the efficacy of medicine at all, and it often happens that in similar cases directly opposite modes of treatment are adopted.

The causes of the small progress in medicine here referred to, Henle considers to be partly subjective and therefore general, and partly objective, i. e. founded on the peculiarities of the object of medicine. That which subjectively or from within ourselves renders experience difficult is, *first,* a tendency natural to the mind to explain and to arrange facts which are seen to recur in inseparable connexion in the relation of cause and effect, and, for physical effects, to invent a world of metaphysical causes. Hence have arisen the mythological fancies of a *materia peccans,* a *vis medicatrix naturæ,* which lie at the bottom not only of the theories of Hippocrates, Galen, Helmont, Stahl, and the so-called natural historical school, but which also constitute the foundation of a physiology still most extensively prevalent. For to define the reaction which supervenes on irritation as a manifestation of an effort of the organism to resist external influences operating prejudicially to it, is merely to declare that every action is a *molimen criticum,* brought about by the autocracy of the organism for the purpose of removing the enemy. Instead of the condition on which the excitation depends, it is merely giving the object or final cause of it. *Secondly,* another tendency of our mind which obstructs the advance of knowledge, is to distort and even falsify facts in order to form systems.

To make medicine, so far as it is not yet, a science of experience and observation is, as is known, the object of the new French school, as the head of which Louis is distinguished. Rejecting uncertain tradition, and aware of the errors which result from coming to a conclusion regarding symptoms and the effects of treatment from the observation and superficial remembrance of a small number of cases, Louis requires that the value of the symptoms, as well as the methods of cure, be expressed in figures obtained by the comparison of as large a series as possible of accurately reported observations. This method, called the *numerical* or *statistical,* is in fact the only one from the employment of which empirical medicine, as well as other empirical sciences, has any advantage to hope for; inasmuch as logical certainty, in decisions founded on experience, is never obtained, but only a greater or less degree of probability, according to the number of observations and the proportion of affirmatives to negatives. Even the so-called *natural laws* possess only the highest degree of probability. In other cases the exceptions are too well established to be denied. The affirmed fact thus loses in probability; it expresses itself no more as a *law,* but only as a *rule* or *norm.* The value of such a rule, however, is variable, and numerically expressible by the proportion of the affirmative to the negative cases. Such expressions are sought for by statistics.

In practical medicine most propositions undoubtedly belong to the disputable. There are few *laws;* as to *rules* they are seldom well established, and when they are, they are of uncertain value. If it must be admitted that in similar cases of disease, comprised under one species, a certain symptom is sometimes present, sometimes absent, that certain phenomena precede often a fatal, but sometimes also a favorable issue, &c., then the necessity cannot be got over for correcting such superficial deductions by means of figures.

Calculations like those above indicated, however, do not warrant more than the expression that the facts stand in connexion according to *law* or *rule;* they teach nothing of their intimate connexion. That such an intimate connexion exists, and in what manner it does exist, it is the theorizing intellect only which shows and elucidates. Sciences of pure experience and observation, therefore, intentionally abstain from giving judgment on this point, and so also does pure empirical pathology. It knows that ulcers of the intestines and typhus fever, swelling of the spleen and intermittent fever, increased quantity of fibrin in the blood, and the local symptoms of inflammation occur together; but it avoids, or ought to avoid, speaking of the causal connexion of these anatomical changes with the symptoms of the disease. It leaves it, for example, to every one's fancy to consider the ulcers of the intestines as the cause or as the effect of the disease. If, however, knowledge, though so empirically acquired, is to attain to practical application, the question as to the causes can no longer be altogether excluded. However superficially the consideration of the phenomena of disease and recovery, considered in themselves, might be allowed to be passed over, the practical application is not at all conceivable, without the presupposition that in corresponding cases a method of cure and the course of a disease had stood in a certain causal relation.

For these reasons it is delusive to believe that in medicine we can rest on a foundation of pure experience and observation. Every inevitable conclusion is a source of logical error, a source of the deceptive "*post hoc ergo propter hoc,*" against which, as is known, the advocates of opposed methods have never failed to warn each other. Medical experience tells merely that treatment and recovery succeed each other; it is inference and hypothesis to say that the recovery is the effect of the treatment. Though this is an old remark it is necessary to repeat it, because it is always forgotten, and the materia medica in consequence overloaded with remedies which are altogether without power of any kind.

The true touchstone for such hypotheses is experiment, and it is the number of experiments which determines the degree of probability with which a certain action is inferred to be the effect of a certain cause, and with which the same effect may for the future be expected from the same cause. But the problem becomes more complicated when many conditions, especially if opposed to each other, have part in one result. If it is desired to learn the value of one of these conditions, for example, a particular method of cure, it is not sufficient merely to count up the cases in which it has been tried with or without success, but it is necessary to give the number of the cases in which cures were effected by it *in proportion* to that of those in which it was not employed, or in which some other plan was had recourse to. This is the object of medical statistics, and for an earnest empiricism there is no other way of coming to a decision—the statistical or numerical method and the empirical are one. Another question, indeed, is what the numerical method has done, and is likely to do for practice. This question will be gone into more fully immediately, but at present it may be observed that when the *numerical method* is not in a condition to guide the choice of remedies, still less so is common so-called experience.

The statistical method is not applicable where there is not at command a considerable number of cases admitting of being compared together. It is not adapted to rare diseases, nor to such as present great irregularities in their course; still less to the not inconsiderable number of chronic diseases, which do not properly belong to any of the recognised families, and are therefore excluded from our system. Its proper field are acute and especially endemic or epidemic diseases, in which the diagnosis is easy and certain, the influence of individual circumstances slight, and governed by the general causes of the disease. But, even in this circumscribed field, statistics are not to be applied without limitation. Acute diseases which, with our present knowledge, we cannot but group together under one head, present themselves according to place and time with very different characters, and it is doubtful, if not positively mischievous, to place observations collected in particular regions and at particular seasons alongside others collected under different circumstances. Acute diseases, which at present are specifically separated, were formerly collected together under one name, and in like manner perhaps our species will themselves be, at some future time, subdivided. Every division of this kind, however, renders the materials hitherto collected worthless. But suppose the identity of the cases of disease under comparison to stand fast, still, how many new difficulties oppose themselves to a uniform action of remedies!

But when, in the class of diseases to which it must limit itself, numerical medicine at last arrives at authentic and stable results by means of a favorable concurrence of all objective and subjective circumstances, objections may still be raised against the practical usefulness of these results. What, says Henle, have we to do when the comparison of two methods of cure gives for the one a slight or even a considerable preference? Are we to adopt it for *all* cases, and are we for the future to make up our minds to the sacrifice of a certain number of patients? Might not those, which we now give up, be rescued by a mode of treatment not adapted for the majority? Are we to comfort ourselves, like gamblers, with the prospect that in the end the gain will exceed the loss? But even if diseases were commensurable, individualities are not so. It is only on the field of battle that fifty living men are an equivalent for fifty dead; to the physician every individual person is incommensurable. The duties, which evidently spring from this circumstance, must be fulfilled by individualizing. But whoever individualizes theorizes. The characteristic of what is individual, in opposition to what is specific, is the concurrence of phenomena occasioned by a multiplicity of disturbing and promoting causes, which does not admit of being prognosticated, and the repetition of which can scarcely even be expected; there can therefore be for the individual neither law nor rule, but there is instead the field of analogy, of combination, of abstraction, i. e. the separation of the accidental from the essential, and the discernment of the import of everything accidental.

To conclude—as pure empiricism does not suffice for every case, and can serve as a guide for our treatment only in a very few,—as we must proceed in particular cases according to particular considerations, which can arise only from opinions on the *signification* of the symptoms and the *nature* of the remedies,—it is a duty to enter as deeply as possible into the nature and intimate connexion of the phenomena which we have to investigate. Whoever is once necessitated to theorize acts foolishly and, if he at the same time undertakes responsibility, unconscientiously, when he leaves it to chance what theory shall occur to him at the moment of decision.

From what has thus been said it will be seen that a strict separation between empirical and rational medicine is not possible.

The *Departments of Medical Science* and the *Position of Rational Pathology* constitute the next subject of the introduction.

The departments of medical science may be divided into three groups: the *first,* comprising what we know of disease, and its different forms, viz. *Pathology;* the *second,* what we know of remedies, viz. *Materia Medica;* the *third,* laying down the rules for the employment of remedies, viz. *Therapeutics.*

Pathology, materia medica, and therapeutics are differently treated of, according as they are studied in reference to the empirical or to the theoretical method.

1st. *According to the empirical method.* In accordance with the way in which the *empirical* physician embraces pathology, he requires true descriptions of the symptoms and course of diseases by which to distinguish them from other diseases. To supply such descriptions is the task of *special pathology,* which may therefore also be named *empirical.* Special pathology views, tacitly or expressly, each case of disease as an individual, combines similar cases to make up the description of the species, collects species into genera, and these into families, &c.; and thus obtains, by an analytical method, a system which, like the systems of the descriptive sciences, discusses the sum of the details.

In the natural sciences, artificial systems have now been completely superseded by natural systems. Medicine seeks to take the same direction, without, however, rightly understanding the requirements for the one or the other kind of classification. Properly speaking, there cannot be an artificial classification of diseases, as there are no signs common to all, and which, with certain modifications of quality or quantity, could be pointed to in every single species. The only exception is as regards duration, according to which, indeed, diseases have been and still are divided into acute and chronic, but that this principle is not applicable for farther subdivision is evident. All other empirical or analytical systems are natural.

The doctrine of symptoms or *semiotics* may be viewed as a complement to special pathology—an index, as it were, to it. Special pathology classifies diseases according to these forms, and lays down the combination of symptoms proper to each species. Semiotics classifies, according to the symptoms, and enumerates the species, i. e. the combinations of the disease in which they appear.

The materia medica is to the empirical physician the table of remedies at his command, with directions for their employment in certain forms of disease or combinations of symptoms comprehended in the system. The action of the remedies on the healthy body is indifferent to him.

The therapeutics of the empirical physician is what is called *special therapeutics.* It points out the approved plan of treatment for every form of disease comprehended in the special pathology. Special therapeutics is to pharmacology what special pathology is to semiotics. Therapeutics indicates the remedies for the diseases, pharmacology the diseases for the remedies.

2d. *According to the theoretical method.* Rational pathology gives an account of the causes and nature of the morbid processes. This used to be done either according to prevailing mythical or "*natur-philosophical*" views, or the characters common to the diseases were grouped together, in order to rise analytically to an idea of the disease. Thus arose the *theory of disease,* or the so-called *general pathology,* in which, besides the specific phenomena of the diseases, special etiology was comprised, and, in consequence of a misunderstanding, semiotics also. General pathology is, however, only a part, and that merely the general part of theoretical pathology.

Rational pathology, such as Henle views it, occupies itself with the vital manifestations of the diseased body. It has, therefore, also been named the physiology of disease, or the physiology of the diseased body. Physiology and pathology are one. If, however, pathology continue separate fron physiology, this is owing merely to the difference in the way pursued by them, and their imperfection hitherto. Physiology sets out from the organs in order to learn the different modes in which their life is manifested under different conditions; pathology, on the other hand, sets out from the manifestations of life, in order, by reasoning backwards to come to a conclusion as to the active organ and the conditions of its abnormal activity. There are symptoms and groups of symptoms to which physiology, in its way, has not yet descended, and to the source of which pathology has not yet risen. Till this be done, there will remain a gap between physiology and pathology, the removal of which is more for the interest of the latter than of the former.

The materia medica of the rational physician is also a physiological doctrine, for it occupies itself in the endeavour to obtain an insight into the mode of action of the remedies on the organic matter. This, however, is very difficult, on account of the therapeutical experiments, from which the conclusions require to be drawn, being much too complex.

If it were but once possible, not only to elicit indications from the nature of diseases and remedies, but also to determine, *à priori,* the mode of procedure adapted to them, we should obtain a rational therapeutics, which comprehends within itself the hitherto so-called general therapeutics in exactly the same way that rational does general pathology. But here also, before planting the field farther is thought of, the weeds already sown by the inconsiderate itch for explanations on the part of those calling themselves empirical must be rooted out.

Rational therapeutics has its special department; and this must be the foundation of the general. Special therapeutics depends on facts partly of rational pathology, partly of empirical therapeutics. Where the former has once penetrated, in the manner already mentioned, to the simple or compound cause of a disease, the prescription for the avoidance and cure of it is evident, viz. to counteract the cause, or neutralize it, or bring back the altered organic material to the normal form and composition. In many cases, however, our knowledge is not sufficient to enable us to lay down rational indications; in other cases, in which such could be laid down, remedies fitted to fulfil them are unknown. Under these circumstances, we take as a fact the empirically established efficacy of certain remedies in certain diseases, and try it with our explanations. This Henle admits to be without doubt the most difficult and as yet the weakest side of rational pathology. A cure is the product of two factors—the disease and the medicine; if one factor is in some degree known, the other may be approximately found, thus from the nature of the organic changes the mode of action of the remedy is inferred, and *vice versâ, ex juvantibus et nocentibus*, the nature of the disease. Unfortunately, however, both factors have in general to be discovered, and in this case different solutions of the question are offered according to the prevailing opinions at the time.

Considering all these difficulties, Henle says that the elaboration of a system of rational therapeutics must perhaps be left to the future.

The third section of the Introduction is on the *Methods of Rational Pathology*.

Rational pathology and physiology are identical; the method of both is therefore the same; it is the method of all sciences of experience and observation, and especially of the natural sciences. By the coincidence of certain morbid phenomena with particular material changes, we are led to the admission of a causal relation between the two; experimenting, we conclude, as far as possible, as to the cause, and assure ourselves, when we observe the results, of the correctness of our conclusion. At the same time, it has in view, first, the so-called localization of the symptoms, i. e. the determination of the organ whence they proceed; but afterwards the quality of the morbid changes by the comparison of the altered form and composition with the normal. The more deeply we penetrate into the finer structure and composition of the tissues, the more manifold, considerable, and important are the differences disclosed. For the most important facts, therefore, pathology is indebted to the microscope and to organic chemistry. The last object is to refer the material processes which constitute the condition of the phenomena of the disease, as far as possible, to physical and chemical processes, and thereby to bring them under a common point of view with the phenomena of inorganic nature.

In the fourth section *the divisions and subdivisions of Rational Pathology* are laid down.

Rational pathology is divided into a general and a special part.

The general part treats of—

1. The idea and nature of disease.
2. The causes of disease in general—general etiology.
3. The general history of disease as regards its locality in the organism, and the mode of its transition from one organ to another.
4. The general history of disease as regards course, duration, and events.

*The special part.* The multiplicity of pathological manifestations is occasioned only by the multiplicity of external causes, and of the tissues entering into the composition of the organs, and the specific forms of disease depend always on the combination of the concrete cause and of the concrete organ. If there was only one cause, there would be as many specific forms of disease as organs; if only one organ, as many forms of disease as causes.

If rational pathology were perfected as a science, the special part would embrace two divisions, viz. special etiology, or the doctrine of the action of the causes of disease on the organs and tissues; and special symptomatology, or the doctrine of the mode of reaction of the tissues and organs. But so long as the specific causes, and the seat of a great many, perhaps the greater number, of diseases are hidden, or at least uncertain, a chapter on the pathological processes, i. e. a chapter in which diseases are arranged according to their symptoms, irrespective of their external and internal causes, is not to be dispensed with.

The fifth and last section of the introduction is occupied with an *Historical Review of Systems of Medicine.*

Into this we shall not enter, but proceed with the remainder of the volume, which comprises the general part of rational pathology, the subjects treated of in which have been just enumerated.

### IDEA AND NATURE OF DISEASE.

Definition. To the ideas *normal* and *abnormal* the ideas *healthy* and *diseased* correspond so far, that the *healthy* is always at the same time the *normal*, the *diseased* the *abnormal*. It is not, however, usual to call everything that is normal healthy, and everything that is abnormal diseased. A crystal which deviates from the normal is not said to be diseased; a congenital defect (or even a defect originating in the course of life), which undergoes no further change, is in general not considered a diseased state. In short, disease is an *abnormal process*, in which there is manifestation of change, progress, or movement. What is abnormal and stationary is a defect,—vitium. The predicate of health, as the opposite of disease, is thus applicable only when there is a capability for an abnormal movement or development. A crystal cannot be said to be healthy but only normal. Health being a movement and a process, it implies the possibility of passing into disease. What cannot become diseased cannot be said to be healthy.

Such is the *idea* of health and disease, so far as it admits of being briefly stated, given by Henle. The *definition* of disease which he founds on it is, "the deviation from the normal typical, i.e. healthy, vital process." This definition, he affirms, is not mere circumlocution. It does not contain, as might at first sight appear, a mere negation, for *the deviating* is;— it is not a *nonentity*, but a *state of being different*. Disease is neither negative, mere absence of health; nor positive, something new, a superstructure on health; but is rather something *instead* of health—a modification of it.

Nature of disease. Fundamentally, disease is—not *abnormal reaction under similar conditions*, but *normal reaction* under different conditions.

This principle, Henle affirms, is either right, or scientific medicine is a chimera. In appearance, indeed, disease is abnormal reaction; pain, as it occurs in inflammation, is an unusual state; but in its nature it is normal reaction, for it is natural for healthy nerves to be the seat of pain on pressure, however caused. It can be surprising only to medical logic, when I affirm, says Henle, that every so-called manifestation of disease exactly corresponds to its external cause. But some one will say, "still irritability is evidently increased in inflammation; the same exposure to cold may cause toothache in one, diarrhœa in another." In answer to this, Henle by the following example illustrates and justifies the meaning of his assertion. In the eye, the sensation of light may be excited, not only by the agency of elementary light, but by other agencies, such as pressure. Suppose, then, common daylight be $=a$, and the direct rays of the sun $=2a$, and that most eyes bear the irritation of $a$ well, but are blinded by $2a$. If an eye be blinded even by $a$, it is said to be abnormal, but if this eye have previously received an impression from some other agency, the excitement occasioned by which corresponds to the mass of light $a$, the sum of irritation which the eye has thus received will be equal to $2a$, and therefore it is not surprising that it should be dazzled as much as the normal eye is by the sun's rays.

In like manner is explained how, by apparently the same cause, different morbid processes are occasioned. In one case, cold operates in secret combination with an injurious influence, which has already altered the vitality of the nerve of the tooth; in another case, in combination with one which has altered the condition of the mucous membrane of the intestinal canal. The product thus appears to be abnormal only when we compare it with *part* of the causes in operation, especially the last; but it shows itself to be normal and necessary, as soon as we take into consideration *all* the causes. The state which in relation to its bearing towards new irritations we call *morbid*, is in relation to previous irritations, the *normal*.

PROCESS OF DISEASE. As all bodies of the same species react in the same way under similar circumstances, the peculiarities of reaction which bodies of different species exhibit under similar circumstances, must at last be referred to peculiarities of type. We have thus to search, in the peculiarities of type which distinguish organic from inorganic bodies, for the cause why it is in the former only that anomalies (or manifestations of the typical force under unusual circumstances) present themselves as *processes*. From the peculiarities of type of organic beings, it happens why it is in them only that anomalies present themselves as diseases or morbid processes. Elimination and renewal of substance is a fundamental character of living beings. It is not wonderful that this fundamental feature preserves itself under unusual circumstances. Only so long as it does preserve itself is the individual diseased; any change which completely stops it, occasions, not disease, but death. Disease is thus a process because life is a process; when it appertains to the type of a body to change its form or substance, abnormal influences change it not only for the instant, but they change also its mode of becoming altered; they turn it away for a longer or shorter time, or for ever, according to the duration of their action, from its aim. The process of development deviating from the aim is the pathological process.

### GENERAL ETIOLOGY.

Proceeding from the proposition that the typical force operates according to unchangeable laws, we must recognise as the cause of every anomaly the action of unusual *external* conditions. The cause of all disease lies originally in an insufficient condition of the compensating material, or in the unappropriateness of the changing influences. Morbid formations of whatever kind are thus originally products of normal typical forces, and inadequate compensating material, or inadequate external conditions of formation. They are merely what the typical force was able to create under the given most unfavorable conditions.

In so far as the body, dependent on external supply, is changeable by external agents, it has the tendency to become diseased. It has health and disease, both *only* in the disposition, because it is a *becoming body*, and because something external must come to it, or be added to it, by means of which it may *become*. The disposition to become diseased is determined by the same typical force as the disposition to healthy development, and is the same for all indivduals of the same species, and existing under similar circumstances of age and sex.

General action of irritants. To the living body is ascribed the property of being excited by every impression to a manifestation of the activity peculiar to it. On account of this property, it is called *irritable* or *excitable*. The influences capable of rousing the slumbering activity are called *irritants;* the operation itself, by which the irritation is responded to, is called *reaction*. Whatever be the irritant, no other reaction takes place but that which is peculiar to the organ.

We view the function of an organ as the consequence of its composition and form, the composition and form again as the result of interchange of substance, the duration and rapidity of which is regulated by the type. Whatever accidentally changes the composition and form, changes the function. The change may have the consequence that the activity becomes quantitatively less or more than it usually is, according to the *tone*, or that slight degree of activity in which an organ, under ordinary circumstances, constantly is without any particular excitement from without. When the activity becomes quantitatively more, the changing influence or irritant *appears to rouse* the activity, supposing it previously overlooked, but, in fact, *changes* it only. What we call irritant, is thus an influence which, at the same time that it alters the substance, changes its powers and activity. But the same thing is effected by every chemico-physical influence on inorganic bodies. The mode in which living bodies comport themselves to irritants is, however, really different from that in which inorganic bodies do to chemico-physical influences; and this difference depends on the fundamental characteristic of living bodies, viz. their property of constantly renewing their substance.

The sort of reaction which is peculiar to the living organism, is that by which the disturbing force is equalized. It is that by which it resists external influences. It is not the excitement which follows the irritation, but the transition to rest *after* the irritation. As two different processes ought not to be distinguished by the same name, Henle retains the name *reaction* for the vital manifestations, which are the immediate consequences of the irritation, and suggests the name *restitution* for the after-action, by which the organ returns to the normal state. From the word reaction the idea of independent reaction, or counter-effect, is thus to be separated; in physiology, *reaction* should mean nothing more than in chemistry, viz. the phenomenon which is called forth in a body by foreign action.

*Exhaustion.* An organ, the action of which is constant, may be quantitatively altered by irritants in two directions; its function may, in comparison with what is normal, appear accelerated and strengthened, or retarded and weakened. The irritants which increase the activity of the function, are named exciting irritants,* irritants in the narrowest sense. The name *depressing* irritants,† although it contains a contradiction, has

* *Hypersthenisants* of the Italian therapeutists.

† *Hyposthenisants* of the Italian therapeutists.

been usually applied to the influences which depress the activity. Both kinds of irritants may operate in such a degree as wholly to put a stop to the nutritive interchange, and for ever deprive the organic structure of its vital endowments, but in different ways: in the case of depressing irritants the complete paralysis occurs *directly;* in the case of exciting irritants, *indirectly.* Both kinds resemble each other in this also, that their effects are, by restitution, gradually equalized; they differ from each other, however, in their remote consequences. After *depressing* irritants, restitution restores the organ to, or nearly to, its normal activity; after *exciting,* it brings it down not only *to,* but *below* its normal activity, to a state of fatigue, from which it only subsequently recovers. The necessity of this latter fact does not appear from what has been above said of the nature of irritation; for if irritation be merely the expression of an alteration of the organic matter, which again becomes equalized in rest, it does not appear why a movement in the opposite direction must first take place. It, however, becomes conceivable when it is remembered that restitution of individual parts, as well as of the whole organism, is dependent on conditions which are furnished to it from without, and the supply of which has no necessary connexion with the cause of the excitement. The individual organs stand in the same relation to the blood as the organism, considered as a whole, does to the vital irritants. With every disposition to react, reaction will not take place if the renewal of the blood be interrupted, or the nutrient constituents of it consumed. On the application of exciting irritants, both results appear to take place; the one by stagnation of the blood, the other by increased affinity of the organ for the vital irritants, or increased nutritive interchange. In consequence of this increased nutritive interchange there is increased energy of function, but, by and by, the source of nourishment becomes exhausted, its supply being limited both as to time and space, the vital conditions thus fail, and diminished energy of function is the result. The contrary is the case with depressing irritants. When an irritant diminishes the affinity of the organic substance for the vital irritants, the nutritive interchange becomes less, hence diminution of the force, hence fatigue and paralysis. The final result of the action of exciting and depressing irritants is the same, viz. diminished vital process, diminished energy, but in the former, from want of new vital irritants, in the latter, from incapacity to receive what exists.

It has been above shown how the excited organ, so long as exhaustion does not ensue, appears to be in a state of increased excitability, because the new irritant concurs with the consequences of a previous irritant. Where qualitative differences are possible, the form of the excitability is at the same time determined by the existing excitement; the organ becomes specifically more irritable for one *kind* of sensations, for the contrasting especially. Here, also, the concurrence of a new change with one already effected in the nerve always occasions the appearance of a reciprocating sum of excitability. The retina, which after green perceives red more vividly, would, after some time, have of itself seen red; it sees, so to speak, doubly red when to the subjective the objective is superadded. On the same principle, a moderate irritant proves a violent one, when it encounters the organ prepared by the contrasting action.

*Exercise and habit.* By exercise an organ becomes more susceptible of irritation, so that smaller quantities are required for exciting it to the same degree of activity. By habit, on the contrary, an organ becomes less susceptible of irritation, so that larger quantities of irritation are required for exciting it to the same degree of activity. This shows that the affirmation, that by restitution the effect of altering irritants is again equalized, is not to be taken literally. It does, indeed, appear that, after irritation, equalization takes place if we view the consequence of a single irritation; but when irritations of the like kind often recur in the same organ, appreciable changes at last appear, which cannot have otherwise arisen than by the addition of similar but *inappreciable* changes after every irritation. The effects of these changes may be either increased or diminished susceptibility.

*Erethism.* In every degree of irritability and tone, the relation, already several times mentioned, of the active organ to the compensating means gives occasion to deviations, which manifest a disproportion between the energy and the duration of the excitement. The rapidity with which the vital excitants are supplied and renewed, is dependent on other conditions than the force with which the individual parts of the body are capable of attracting it. In general both increase simultaneously, but there is a state possible in which the capacity for renewal lags behind excitability, to this state the name of *erethism,* or *irritable weakness,* is applied. It is especially remarkable when the excitability is normal, or even more vivid than usual.

Erethism is local or general. An example of local erethism is presented by parts, the vascular system of which has a particular disposition to congestion. Examples of general erethism are met with after great losses of blood and other fluids, and in convalescence from febrile diseases.

*After-action.* It has been admitted that every irritation changes the relation of the irritated part to the blood; in accordance with this, the condition of the blood must, according to the deviations of the states of excitement of individual organs, be modified, and hence, as the blood is the common source of the nourishment of all organs, every local change must at last induce a change of the whole organism. This change may be, to a certain degree, either beneficial or prejudicial; beneficial as when, by appropriate exercise, the general condition is improved along with an increase in the power of the exercised organ and in the appetite; prejudicial, as when the loss of the vital irritants is not at all, or not rapidly enough supplied, and general exhaustion produced, the primarily-excited organ itself being at the same time weakened, or, on the contrary, strengthened.

Disposition to disease. In its relations with the external world, in its dependence on the vital irritants, in its changeability by accidental irritants, the organism possesses the *capability* of becoming diseased. In order that disease may *actually occur*, the operation of external influences on the organized individual is necessary. The particular disease which shall occur depends on the particular organic relations and mode of reaction of the individual, and on the concrete nature of the external influences.

An external and an internal condition must thus concur in the production of a particular form of disease—the internal condition constitutes what is called *predisposition to disease* (*seminia morborum* of Gaubius).

The admission of particular predispositions rests on the fact that the same injurious influence produces, in different individuals, different diseases; that it sometimes acts violently, sometimes mildly, or not at all.

A different effect of a similar cause, however, does not and cannot occur in individuals of the same species, as above shown. When there is an appearance of such a thing, it is found, on examination, that either the individuals compared existed under different conditions of normal formation, or under the influence of different external agencies. It is necessary to distinguish these two cases; for the individual which reacts peculiarly only by virtue of the sex or age to which it belongs, is healthy; the individual, on the contrary, which, on account of co-operating external influences, manifests a particular disposition, is already changed by such influences, and is therefore diseased. The disposition which depends on the normal relations, Henle calls *normal predisposition,* and that which depends on the previous operation of external influences, *abnormal predisposition.* The former only is really innate—the inheritance of the race as it came from the hands of the Creator; the latter is always acquired, though to the individual in which it appears it may have been transmitted by generation.

*Normal predisposition to disease.* To the normal conditions on which peculiarities of predisposition depend, belong age, sex, perhaps race, temperament, and similar individual characters within the norm. From the comparison of the peculiarities of reaction occurring in one species, genus or higher class with those of other species, genera, &c., the normal disposition of the group in question is ascertained.

The comparison of different predispositions may lead to two results:

1. That the disposition to become diseased in general may be greater or less according to age, sex, race, &c.

2. That certain forms of disease exclusively or especially belong to a particular group.

In the first case we distinguish the *quantity,* in the second the *quality* of the predisposition.

In regard to the quality of the predisposition: the forms of disease are, under similar external circumstances, directly determined by the nature of the organs and tissues acted on by the cause, and the organs and tissues being the same, by their peculiar typical modes of reaction. The tendency to become diseased in a particular manner, or the *qualitative* predisposition to disease, is thus dependent on the constitution of the organs and systems, and on the modes of reaction.

*As to the constitution of the organs and systems.* It is self-evident that every group is distinguished by anomalies of the organs which are exclusively peculiar to it; that the non-development, arrestment of the development, and certain malformations present themselves as diseases only at the age at which development is not yet completed, and still to be expected, or are first perceived at the age when the organ should enter upon its function. From this it is seen how that systems anatomically or functionally predominant must enter into manifold relations with the external world and the other organs; and be exposed in an especial manner to noxious influences. In this way is explained, the correctness of the fact being assumed, the very great tendency to diseases of digestion in childhood, of the lungs in youth, and of the mind in manhood.

*As to the modes of reaction.* By nothing but by a reference to the eternal typical laws is it to be explained why in the salamander whole members are reproduced, whilst in the allied frog, regeneration is, as in the higher animals, limited to a few tissues ; why the same kind of wound in one species of the mammifera heals with the formation of a crust, in another by suppuration ; why, lastly, the same substance is in one species harmless, in another poisonous.

*Abnormal predisposition to disease.* Abnormal predisposition is itself, in fact, disease. To it is applicable the expression "*affectio,*" with which Gaubius designated predisposition in general. The pathological state called predisposition may be undeveloped, and as yet without manifest symptoms, *affectio occulta* of the older authors ; or is already evident disease, but preparatory (*affectio predisponens*) to another state of disease, which is either a higher degree of that which already exists, or a disease different in quality from it. Morbid predisposition has, moreover, this in common with disease, that it may be persistent or temporary, inherited or acquired after birth.

*Pars minoris resistentiæ* is an organ which is habitually in a state of over- or under-excitement, in comparison with the other organs, and assailable from all points of the body by every kind of influence. In a person with a carious tooth, for example, toothache may be brought on by cold, by heat, by mental affection, errors of diet.

*Hereditability.* From fissiparous generation literally, from gemmiparous generation very evidently, and oviparous generation not less really, though less evidently, it appears why the action of morbidly altering influences may extend to the posterity, nay, that they *must* extend. But they do not explain why specific diseases, such as dyscrasiæ of the parent are repeated in the offspring. Disease thus transmitted must be distinguished from heritable or inherited disease, which is not merely a continuation but also a repetition of the disease of the parents.

Hereditability of disease is no farther explicable than by reference to the empirical law that the young resemble their parents in their peculiarities.

Hereditary disease may already exist at birth (*congenital*), or make its appearance only after birth. When not congenital, it is to be viewed as *affectio occulta* until the period favorable to its outbreak. Congenital hereditary diseases are not to be confounded with diseases which, though congenital, have had their origin in some accidental cause during intra-uterine life.

*Constitution. Habit.* The name morbid predisposition has been above employed to designate every permanent or temporary, slight or inveterate abnormal state, in so far only as it is viewed as preparatory to some other morbid state. The name *constitution* is employed to designate a peculiar kind of predisposition which is in any case permanent, mostly congenital or inherited, and in no case important enough to be viewed as a disease in itself. The state which is comprehended under the name of *predisposition,* as something relative in reference to a certain morbid process, appears, under the name of *constitution,* as something absolute, ready, and of itself existing. Predisposition and constitution are apt to be confounded; for example, scrofulous predisposition instead of scrofulous constitution. Scrofulous constitution is that kind of constitution in which the predisposition to scrofula exists. The constitution is, as mentioned, generally congenital, and at any rate, developed by subsequent influences; still it may be also acquired, or, which is the same thing, qualitatively changed by the mode of life and supervention of disease.

The external sign of the constitution, or of a permanent anomaly which cannot yet be called disease, is *habit*. The elements which compose the habit are the form and colour of the exterior of the body, and the condition of it, from which a conclusion may be mediately drawn as to the nutrition, the quantity of blood, the richness of the blood in particular constituents, the energy of the nervous and muscular systems, &c.

EXTERNAL CAUSES. So long as the combination of symptoms, on which the diagnosis is founded, was considered as an entity and called disease, the internal derangement, which is the condition of the symptoms, was spoken of as the proximate cause of the disease. This *causa proxima* was distinguished from the remote causes, *causæ remotæ*, the causes of the derangement itself. Nowadays, when symptoms are no longer viewed as manifestations of an ideal entity, but as the effects of the material derangement, this derangement is recognised as the disease itself.

A great, perhaps the greatest, part of diseases which come under observation is the result of a plurality of causes. When several causes occur at the same time, a threefold consequence is possible: the effects of each cause may—1st, proceed in company, little or not at all disturbing each other; or, 2d, increase each other; or, 3d, limit each other; or, 4th, neither increase nor limit; but there may result from their combination an effect qualitatively different from any of them.

### LOCALITY OF DISEASE, AND MODE OF TRANSITION FROM ONE ORGAN TO ANOTHER.

SYMPTOMS OF DISEASE. The healthy state of the body and organs is recognisable by the external appearance, the form and composition of the parts, and by their functions. The morbid state is manifested by deviations of these from the normal condition. Changes of the aggregate state and function, by which the disease is made known to the patient himself, or objectively to others, are called the *symptoms* or *phenomena* of the disease.

Strictly speaking, symptom is thus the external morbid phenomenon, whether changed function or change of aggregate state. Disease, on the contrary, is the material derangement which is inferred to be the cause of the morbid phenomenon. According to this, symptom is the *occasioned*, disease the *occasioning;* and to symptoms as well as to disease there belong two attributes, viz. *symptom* is the external and the effect, *disease* the internal and the cause. Symptom considered as the external sign of the disease, though always in causal relation to it, is not always its direct effect, but may be merely the effect of a direct effect of the disease, thus headache is not the direct effect of derangement of the stomach, but is the effect of some change in the frontal nerve, which is the direct effect of the derangement of the stomach.

On the other hand, symptom considered as the effect, is not necessarily external sign, but may be the cause of external signs; thus effusion of serum into the cellular tissue, which is a symptom of certain diseases of the blood, of pressure on veins, is not itself an external sign, but is the cause the external signs of dropsy.

*Symptomatic and idiopathic disease.* Jaundice may arise from disease of the liver occasioned by disease of the stomach, brain, or other organ, or it may arise from primary disease of the liver. In the former case, it is said to be symptomatic, in the latter case idiopathic. In both cases, however, the jaundice is merely a symptom of the liver complaint, which, in the former case, is the symptomatic disease, in the latter, the idiopathic.

The symptoms of the cause of the disease are to be distinguished from the symptoms of the disease itself; for example, the smell of spirits in the breath of a drunk person is the symptom of the cause of the drunk state.

Local and general diseases. The determination of the general or local nature of a disease was not possible until it was determined what is meant by disease. Fever was, at one time, said to be general, because it was considered to be a disease of the blood; but when the change in the blood was found to arise from a local cause, fever came to be viewed as a local disease. When, however, the local cause was discovered to be the effect of a deterioration of the blood, fever was again viewed as a general disease. In all this the true proximate cause of fever was not yet taken into consideration. Had it been remembered that the febrile symptoms are owing to an affection of all or many nerves, the disease would have been viewed as general; and had it then been found that the cause of the affection of the nerves lay in a part of the central organ, fever would have again been put down as local.

We now know that disease is a process comprising different changes, partly local, partly general—changes, the causal relations of which with each other may be very manifold. Before a disease is put down as local or general, it must be determined which link in the chain of its conditions is the most essential, and, above all, in what mode of connexion the links stand to each other in individual cases.

As every drop of blood is mixed with the whole, if a drop be altered in any part of the vascular system, the alteration is communicated to the whole. Disease of the blood is, therefore, necessarily and absolutely general, and may be contradistinguished from diseases of the solids, which are properly local, and only more or less disseminated. This distinction, however, is of little value, as few diseases remain confined either to the fluids or to the solids.

General disease, in the empirical sense, requires to be distingished from general disease in the theoretical sense. General disease, in the empirical sense, is the proximate organic condition of the general symptoms. General disease, in the theoretical sense, is a disease of the blood alone.

*General* disease, or disease from general causes, is frequently understood in the same sense as *constitutional* disease; a *constitutional* or *general* predisposition is presupposed in the case of a symptom which appears to have arisen without sufficient occasional cause. This confusion may, in most cases admit of justification; most constitutional diseases are not only in the empirical, but probably also in the theoretical sense, general, seeing that they may break out, not only in many parts of the body, and in the whole extent of a tissue, but also appear to owe their first origin to such causes as operate on and through the juices. It is, however, a too hasty proceeding, and one not without disadvantage to the investigation of the individual pathological processes, to assume constitutional disease and disease of the juices as exactly synonymous. Every disease of the juices is not constitutional, nor is every constitutional disease a disease of the juices. Madness and rheumatism may be constitutional, but are they owing to a morbid condition of the blood?

That disease is constitutional which arises from a long series of inconsiderable influences without marked, or, at least, without adequate occasional cause. General diseases are diseases of the blood without reference to origin. Syphilitic dyscrasy Henle would call general, not constitutional syphilis. Of hereditary mental diseases, he would say that they arise from internal or constitutional, not from general causes. Other diseases, as gout, scrofula, &c., are, at the same time, both constitutional and general.

SYMPATHY AND ANTAGONISM. The various parts of the organism co-operate in the maintenance of the whole; each part thus stands in reciprocal relation with all the others, and continues healthy only so long as all the others perform their functions correctly. If this relation of all the parts to each other were general and uniform, the result of the change of one must be equally communicated to, and spread over all the others. There is, however, both in physiological and in pathological processes, a particular co-operation between particular organs, so that the changed condition of one is expressed by a changed condition of another, and excitement of one occasions excitement or, its contrary, diminished activity of another. Strong irritation of the retina, for example, occasions contraction of the pupil, closure of the eyelids, and sneezing; disease of the liver, pain in the right shoulder.

Two organs which are thus excitable and changeable by each other, stand, as is said, in sympathy or consensus.

There are two modes of sympathy, viz. synergy and antagonism. The name synergy, Henle proposes for that mode of sympathy in which the same change ensues in one organ which was excited in the other, and the name antagonism for that mode in which the change in the first organ occasions an opposite change in the second. In general, the sympathetical connexion between two organs may be either exclusively synergical, or exclusively antagonistic, or alternately synergical and antagonistic, and perhaps only more frequently in the one way or in the other.

What the conditions are, on which synergy in one case and antagonism in another depend, have not been made out except in a very few cases.

The phenomena of sympathy, as well synergy as antagonism, depend on physiological grounds—on the original normal connexion of individual organs and tissues. Those communications only are sympathetic which are conveyed by a permanent and pre-existing relation of the parts to each other. There are, however, normal and abnormal sympathies. Normal sympathies occur in every healthy body, as they are partly conditions without which the normal function would not be exercised. Sympathies are abnormal, either in consequence of the readiness with which, or the extent to which they take place, or in consequence of their occurring between parts which do not usually sympathise. The organs between which abnormal sympathies occur are pathologically changed.

According to the place where the cause of the disease has operated, the sympathies define the extent and form of the disease. The special knowledge of them is indispensable to the practitioner, partly for the sake of diagnosis—for the symptoms of many diseases of internal organs are the consequences only of sympathetical irritation in structures more accessible to exploration; partly for the sake of therapeutics, for many organs can be affected only through others connected with them sympathetically. On the laws of antagonism is founded the treatment by derivation.

The formula which may be established as the pretty generally applicable expression for the process of sympathy is: A *acts through the medium* X *on* B. The communicating medium is the *source* or *conveyer* of the sympathy.

It has been above observed that the idea sympathy or *consensus* comprises the idea of a permanent connexion originally existing in the organization. As the carrier of sympathy, therefore, the permanent structure comprehended in the original plan of organization is to be recognised.

In short, sympathy may be defined the connate or permanent connexion of the parts of an organism kept up through the medium of the normal tissues or organs in such a way, generally reciprocal, that the changed state of the one has for its consequence a changed state of the other.

This definition Henle considers to be in general sufficient to distinguish sympathetic reactions from those which are direct—the simply consecutive—and from those which arise from the extension or transplantation of the cause, or from accidental morbid products.

As communicators of sympathies two parts are known, viz. the blood and the nervous system. Accordingly, two classes of sympathies are recognised: sympathies through the blood and sympathies through the nervous system. To these must be added a third class, in regard to the source of which, however, it is not certain whether it be through the blood or the nerves.

A. *Normal Sympathies.*

I. *Sympathies through the blood.* Sympathy through the blood is presented by those parts which have similar relations to one of the constituents of that fluid,—in nutrition, in secretion, and in excretion. If the particular constituent be increased in quantity in the blood, the action of all the parts in question is increased. The constituent remaining the same, inactivity of one of the parts will call forth increased activity of the others, and *vice versâ*. Sympathy through the blood thus always presents itself in the form of antagonism. Examples of antagonism through the blood are presented by the skin and kidneys as regards the excretion of water, and between the lungs and skin perhaps as regards carbonic acid.

II. *Sympathies through the nerves.* The communication of excitement from one nervous fibre to another in the central organs of the nervous system, viz. the brain, spinal marrow, and ganglions, is the basis of all unequivocal sympathies through the nerves. The laws according to which the communication is effected are at the same time the laws of sympathies.

It is impossible to conceive the communication to be effected in any other way than that of proximity in space. Nerves which diverge peripherally may be in close proximity in the central organ, hence distinct parts of the body may be in such sympathetical connexion, as if the irritation immediately spread from one to the other.

If our knowledge of the arrangement of the origins of the nerves in the central organs were perfect, nothing farther would be necessary than to show that nerves which are here in contact stand in special sympathy with each other. We are, however, still far from possessing such a knowledge of the structure of the nervous system.

The sympathies between the peripheral parts of the body are better known, and if it were only proved that sympathetically excitable nerves lie together in their central course, more certain conclusions might be drawn from the sympathies as to the structure of the central organ. The proposition therefore, that nervous sympathy is the consequence of an extension of the excitement in the central organ according to the contiguity of the nervous fibres, is not to be taken for more than an hypothesis; an hypothesis which will be found the more admissible, the more frequent, on the one hand, sympathies between reputed neighbouring nervous fibres are, and on the other, the more it admits of being rendered probable that nerves sympathetically connected approximate each other by their central ends.

The conjectures as to the arrangement of the nervous fibres in the central organs are founded partly on anatomical, partly on physiological researches. As a basis, therefore, for his discussions on the nervous sympathies, Henle devotes a considerable space to anatomical and physiological observations on the nervous system, which, however, we must pass over, and then observes that, in order to separate as much as possible what is certain from what is hypothetical, he will treat of the sympathies of each group of nerves separately; enumerating in each successive group, the sympathies of the nerves belonging to it with each other and with the nerves of the preceding group. Sympathies are divisible into five classes: 1. Sympathies of recognised cerebro-spinal nerves, i. e. of the sensitive nerves of the external parts of the body, and of the voluntary motor nerves, with the exception of the sensorial organ. 2. Sympathies of the visceral nerves, sensitive and motor. 3. Of the nerves of the cellular tissue. 4. Of the nerves of the vessels; and, 5, of the psychical nerves.

1. Sympathies of the cerebro-spinal nerves. There are three directions in which a communication may take place through the central gray substance of the spinal marrow, between the fibres of the white cords, viz. 1. Broadways, or from one side to the other. 2. Lengthways, from below upwards, or from above downwards. 3. Thickways, or from behind forwards, or from before backwards. Thus, if a sensitive nerve be excited from without, the excitement is directly communicated either to the corresponding symmetrical sensitive nerve of the other side, or to a sensitive nerve of the same side immediately above or immediately below, or, lastly, to the motor nerve of the same side situated on the same level. To one or other of these three kinds of communication all sympathies may be referred.

Synergical communication:—

From one side to the other. *a. Sensitive Nerves.* The following are examples: The supervention of toothache in the sound tooth of the opposite side. The case observed by Ollivier, in which, though the skin of the left side of the body was itself insensible to pinching, &c., a slight sensation was thereby excited in the skin of the opposite side. The fact that the width of the pupil is regulated in each eye by the sum of the light falling on both, as shown by this, that if one eye be closed, the pupil of the other will become somewhat dilated, though still exposed to the same degree of light it was individually exposed to before; and that when one eye is amaurotic, the pupil is dilated while the sound eye is covered, but generally contracts when the sound eye is opened to the light. This, Henle thinks, may be owing to sympathy between the two retinæ, or to an association between the motor nerves of the two irides. *b. Motor Nerves.* As to synergical communication from one side to the other between symmetrical motor nerves,—voluntary nerves at least,—Henle thinks it difficult to say, whether the following, out of several others, be a real example: a case of hemiplegia recorded by Dr. Marshall Hall, in which movement of the sound arm was accompanied by unconscious movements of the paralysed one.

As to the more compound diseases which affect symmetrical organs or regions, Henle observes, that in many cases symmetrical disease is referable to the similarity of the conditions of the two organs as regards structure, exposure to cause, and especially as regards the condition of the blood. But why particular symmetrical regions of the skin, particular joints, &c., become diseased in preference to other regions of the skin, other joints, &c., of the same side, Henle thinks must rather be looked for in the nervous system.

Ascending and descending communications. *a. From sensitive to sensitive nerves of the same side.* The facts that almost every violent pain apparently extends in the neighbourhood of the affected part, that strong excitement of the retina occasions tickling in the nose, that a disagreeable sound sets the teeth on edge, and produces creeping of the skin, &c. may be viewed as examples of this kind of communication.

*b. From motor to motor nerves of the same side.* As examples of this kind may be mentioned: difficulty of moving certain fingers without others. Such cases as these, a patient with paralysis of the left side, when he yawned raised the paralytic arm; another patient when he sneezed raised the paralytic arm, and when he coughed, the paralytic leg, &c. Many spasmodic diseases, as squinting, stuttering, &c., depend on a morbid tendency to such sympathetic movements.

Communications from before backwards, or from behind forwards. *a. From before backwards—from motor to sensitive nerves.*—Examples: *Reflex sensations.* The common case of cramp and neuralgy. *b. From behind forwards—from sensitive to motor nerves.* Examples: *Reflex motions.* The best known and most extensive sympathies.

Antagonistic communication:—

The most striking examples of antagonism are presented by the anterior and posterior columns of the spinal marrow in their reciprocal relations. Irritation of a part of the skin occasions, by synergy, reflected cramps in corresponding muscles. Cramps originally owing to internal causes, on the contrary, admit of cure by irritation of the corresponding sensitive nerves; it is well known that painful cramp of the calves of the legs may be relieved by rubbing the skin. To reflex sensation, in which cramp occasions pain, stand opposed, as corresponding antagonistic phenomena, those cases in which muscular action depresses the sensibility.

2. Sympathies of the visceral nerves. *a. Synergy.* In regard to the visceral nerves, the preliminary anatomico-physiological investigations led to the conclusion that they end partly in the ganglions, and partly in the spinal marrow, after having passed through the ganglions, and that, before joining the spinal marrow, they usually run in the limitrophic cord for some extent upwards, in order to join a higher situated spinal nerve ; more rarely downwards, to join a lower situated spinal nerve. As to the motor nerves of the intestines, it remained undetermined whether they extend into the brain ; as to the sensitive nerves. Henle believes that it may be affirmed that they do extend into the brain. If this view be correct, and if the gray substance of the ganglions, as well as that of the brain and spinal marrow, is a conducting medium, smaller tracts of the intestines must be brought into sympathy through the ganglions in and on the organs, whilst larger tracts of the intestines must be brought into sympathy through the remoter and larger ganglions into which many nerves enter; and, lastly, whole tracts of the intestines, and the intestines themselves must be brought into sympathy with the external parts of the body through the spinal marrow.

The results of experiments on reflex motions correspond with this construction. Henle observed in mammifera and frogs, that the circular contraction which ensues on superficial irritation of a circumscribed region of the gut was limited to a very small extent, when the gut was cut off close to the mesentery; that, on the contrary, the contraction extended to the neighbouring parts in a progressive, peristaltic manner, when, after destruction of the brain and spinal marrow, the connexion of the nerves of the gut with the ganglions was left undisturbed; and, lastly, that the most extensive peristaltic motions took place when the gut was locally irritated in recently slaughtered and irritable animals in which the central organ had not been injured. When organs wholly removed from the body respond to a partial irritation by movements in their totality, especially by regular movements, the conducting ganglions must be contained in the substance of the organ, as has been proved in regard to the heart, and rendered especially evident by Volkmann's experiment, in which, after the heart has been divided longways above a certain point, its synchronism is destroyed, and under certain conditions also the propagation of the excitement from one half to the other.

In order to produce reflected motions in the external parts by exciting the intestines, the spinal marrow must be entire, and in the frog the medulla oblongata also.

From the sympathies of the intestinal nerves *with each other*, reflex movements and reflex sensations are, in pathological and physiological processes, especially remarkable. Under the head of reflex sensations may be reckoned the pain in colic and during uterine contraction. As in external parts, reflex motions take place in general immediately under the part of the skin irritated, so reflex motions ensue on irritation of serous membranes as well as mucous, and that, as Henle believes he has remarked, more readily in the first than in the second case. Still certain regions of the mucous membranes also stand in relation with remoter muscles: thus, when there are worms in the intestinal canal, the pupil becomes dilated in consequence of the irritation of the sympathetic root of the ophthalmic ganglion.

In the relation of the intestinal to the external nerves, there appears, in the first place, to exist a sympathy between the walls of the cavities and the contained organs; for example, not only between the scalp and the brain, between the skin of the neck and the larynx in which there might be suspected a vascular communication, but also between the walls of the thorax and lungs, between the walls of the abdomen and the viscera.

Sympathetic sensations. The following are common examples of sympathy. If the stomach is disordered we have headache; in disease of the liver, pain in the right shoulder; in disease of the spleen, pain in the left shoulder; itching of the nose and anus in worms; itching of the glans in nephritic calculi. In most cases in which external nerves participate in the state of excitement of internal, the pain in the former is the most prominent symptom, because, in regard to the situation and quality of pain in the latter, the consciousness is less definite. For the same reason, on the other hand, communication from spinal to sympathetic nerves is rarer.

Sympathetic motions. Involuntary muscles very frequently come into play during the action of voluntary muscles, thus the *iris* contracts when the eyeball is turned in, and what deserves to be especially remarked is, that the inverted position of even only one eyeball, or voluntary squinting inwards with one eye, has the effect of determining contraction of the iris of both eyes,—a sympathy, the ground of which cannot be well explained, except by a reference to the cerebral origin of the motor nerves of the circular fibres of the iris. As other examples of sympathetic motions, it may be mentioned that in laughing, sympathetic contractions of the abdominal viscera may occur, occasioning involuntary discharge of urine or flatulence. The sympathetic vomiting in coughing is well known. The muscular walls of the bladder, uterus, seminal vesicles, intestines, may be excited to action by voluntary contraction of the abdominal and pelvic muscles. The palpitation of the heart, which occurs in consequence of muscular exertion, is referred by Müller to the head of sympathetic motions, and Henle explains in the same manner the contractions of the bronchi and the necessity for accelerated respiration in ascending hills, &c.

Reflex sensations. Henle admits that there is scarcely an unequivocal example of reflex sensation between internal motor and external sensitive nerves, or between external motor and internal sensitive nerves.

Reflex motions from nerves of the trunk to internal nerves, and *vice versâ*, are the most frequent. Besides the cases, daily met with, of cough, hiccough, and vomiting from affection of mucous and serous membranes, there may be mentioned, as proofs of the peculiar relation between higher external and deeper internal organs, squinting in disordered states of the stomach and in worms, trismus from worms, as also, in the contrary case, vomiting from irritation of the optic or trigeminus nerve produced by pressure on the eye and in headache, &c.

2. Sympathies of the visceral nerves. *b. Antagonism.* Abercrombie first showed that in ileus there is not spasm but paralysis of the gut, which paralysis comes on sometimes alone, sometimes in connexion with inflammation of the mucous membrane; in the latter case the inflammation may have been present from the first, or have only come on subsequently. There is thus, says Henle, if we regard the appearances only, antagonism between the mucous and muscular coat—irritation of the former with paralysis of the latter. But another question arises, viz. whether the intimate connexion is here that of antagonism, whether the paralysis is occasioned by the irritation of the mucous membrane operating through the nerves. Henle thinks that the same disturbance of the circulation which is the cause of the symptoms of inflammation in the mucous membrane, at the same time directly depresses the tone of the muscles.

In neuralgia of the fifth pair there is often mydriasis. If this be owing to paralysis of the circular fibres of the iris, the nerves of which come from the third pair, the case would be one of antagonism; but if the dilatation of the pupil be active, and owing to irritation of the sympathetic root of the ciliary ganglion, the case would be one of synergy betwixt this root and the sensitive fibres of the fifth pair. Facial neuralgia and cardialgia often alternate with each other.

3. Sympathies of the cellular tissue. The sympathies of the cellular tissue are limited, like those of the vessels, to sympathetic motions, reflex motions, and perhaps antagonistic paralysis.

As the iris moves in concert with the muscles of the eyeball, so the dartos contracts in concert with the contractions of the muscles of the perineum, and even of the lower extremities. Violent contraction of the sphincters of the anus and bladder, in order to resist the tendency to evacuation, has also for its accompaniment corrugation of the scrotum.

Cramp of the skin (*cutis anserina*) from disagreeable sounds and cold, is an example of reflex motion.

In local irritation of sensitive nerves, and local reaction of the cellular tissue, the cramp manifests itself at the irritated part of the skin: e. g. erection of the nipple, and contraction of the dartos from tickling of the superincumbent skin, bristling of the hair in violent neuralgia of the head.

4. Sympathies of the nerves of the vessels. The tone of the vessels admits of being inferred only mediately from the resistance which they oppose to the blood sent from the heart. The force of the heart and the quantity of blood being given, each vessel will yield to the wave of blood the more readily, and will be by it the more dilated the less resistance its muscular tunic offers. The distension may be immediately perceived either by the sight or touch in the larger vessels towards the surface. Strong pulsation, so far from being a sign of increased activity of an artery, is a proof that it is in a state of relaxation. The distension, and mediately the power of resistance of smaller vessels, may be inferred from the colour and turgescence of the tissues; for the broader the streamlets of blood in proportion to the parenchyma, the more does the red colour of the blood prevail; and the wider the vessels, the thinner their walls, and the more copious the exudation through them, which is indicated by swelling or effusion of plasma. In glands, increased secretion takes place in this way; in other parts, the symptoms of dropsy are developed, inflammation and its events. On the other hand, contraction of the vessels occasions paleness of the tissues, collapse, and, when the state continues, atrophy. Under certain circumstances when the arteries are contracted, the blood accumulates in the radicles of the veins, and occasions a blue, livid colour of skin, which may easily be distinguished from the redness owing to capillary congestion.

We are thus in a condition, due reference being made to the power of the heart, and the quantity and condition of the blood, to diagnosticate, in individual cases, the state of excitement of the nerves of the vessels. And as both conditions are always the same for all parts of the body, so the comparison of the colour and turgescence of any one part with those of the others, gives us the measure for the actual excitement of the vessels of any one part.

This excitement becomes changed along with the activity of the organs, whether sensible or motor : it rises or falls with it in a direct or inverse ratio.

*a. Synergy.* 1. *With motor nerves.* In most if not all cases of muscular contracture, there is at the same time continued spasm of the blood-vessels of the part, not of the muscular branches merely, but also of the trunks and vessels of the skin covering the muscles. 2. *With sensitive nerves.* Sometimes in *tic douloureux,* and also in commencing inflammation ; paleness precedes the redness, in other words, contraction of the walls of the vessels precedes relaxation.

*b. Antagonism.* 1. *To motor nerves.* This is manifested by the increased nutrition of a part much moved. In the active muscular exertion the vessels become relaxed, and therefore dilated, the consequence of which is increased exudation.

2. *To sensitive nerves.* Examples of this are :—increased excitement of sensitive nerves with diminished excitement of the nerves of vessels, and diminished excitement of sensitive nerves with increased excitement of the nerves of vessels. Increased excitement of sensitive nerves with diminished excitement of the nerves of the vessels, and consequently the dilatation of the vessels, is manifested by the symptoms of inflammation.*

Increased excitement of sensitive nerves from internal causes has also diminished excitement of the nerves of vessels for its consequence, as is shown by the flow of saliva, tears, and nasal mucus, by the injection of the conjunctiva, &c. in facial neuralgia, &c. Increased excitement of special nerves of sense is perhaps also a cause of diminished action of the nerves of the vessels, thus undue exertion of vision is followed by congestion of the eye. As an example of diminished excitement of sensitive nerves with increased excitement of the nerves of vessels, may be mentioned the contraction of vessels which, in cold, occurs in company with diminished sensibility.

The sympathy of animal and organic nerves, whether manifested as synergy or antagonism, presents itself as regards locality quite in accordance with the phenomena of reflex action.

5. Sympathies of the sensorial organ. The connexion betwixt the nerves of the body and the sensorial organ manifests itself sometimes as synergy, sometimes as antagonism. A thought, with passion, has for its consequence sympathetic movements and phantasies of the senses : the activity of the sensorial organ may, however, be so concentrated in the thought, that the motor nerves sink even below the average of their excitement,—below the normal tone. During intense contemplation of an object, the energy of the muscular nerves is diminished, as manifested in the features of the face by a paralytic-like expression ; and in passion the members may refuse their office and the sphincters relax. On the other hand, bodily activity depresses the liveliness of the psychical affection ; anger and vexation, sorrow and joy become worn out in the half involuntary muscular action, in the spasmodic respiratory movements which they first sympathetically gave rise to.

* See Report on Inflammation, in this Review, for April, 1844.

A direct communication between the sensorial organ and the nerves of vessels is not demonstrable. When in mental affections the tone of vessels is changed, there are always at the same time altered sensations, and also frequently unusual muscular action, and on these the condition of the vessels may immediately depend.

III. *Sympathies, the source of which is unknown.* Under this head Henle places,—1, the sympathy betwixt the breasts and the uterus, including the ovaries, by means of which irritation of one or other of these organs operates sometimes synergically, sometimes antagonistically, on the sympathetically connected organ. The breasts swell and are painful after conception (synergically), but also after delivery, and sometimes even after the death of the child (antagonistically); although in the latter case they more frequently become flaccid; lactation generally prevents menstruation, and limits the capacity to conceive, and if during lactation it should so happen that menstruation or conception take place, the awakened activity of the uterus puts a stop to the function of the breasts. Blisters on the breasts excite menstruation (synergically), and stop discharges of blood from the uterus (antagonistically). 2. The sympathy betwixt the parotid and the breasts and the ovaries in the female, betwixt the parotid and testicles in the male. Rheumatic swelling of the parotid transfers itself to the organs mentioned, and may, by means of irritants applied in the region of the parotid, be again brought back to this organ.

B. *Abnormal Sympathies.*

I. *Abnormal sympathies through the blood.* Henle gives the following illustrations of the conditions under which the blood may become the medium of an abnormally acquired sympathy:—1. Suppose the blood to contain an abnormal excrementitious matter, for the excretion of which the skin was delegated, and suppose, moreover, that the kidneys or any membrane possessed the power to attract and deposit that matter when it remained in the blood, then suppression of the cutaneous secretion would be injurious in a manner quite different from that in which it is usually injurious, and the organs which would suffer from the derangement of the function of the skin would be different from those which usually suffer. 2. A new antagonism is established in chronic dropsy, between the affected serous cavity and the kidneys, on which the cure of dropsy by means of diuretics depends; in gout, between the parts of the body which are the seat of the deposition of uric acid and the kidneys, so that the formation of gouty deposits alternates with gravel and urinary calculi.

These cases of morbid sympathy, however, are rare and doubtful.

II. *Abnormal sympathies through the nerves.* When a smell excites nausea or venereal desire, it is not because there is any particular sympathy between the nose and the stomach, or between the nose and the genital organs; the effect is produced through the mind. Again in persons disposed to toothache, overloading of the stomach will bring on an attack, not, however, because a sympathy exists between the stomach and teeth, but because the overloading of the stomach excites all the nerves, and the state of excitement of the nerves of the teeth already existing is readily increased to pain.

The external conditions on which the occurrence and spread of the communication of sympathy depends are the strength and kind of the irritation. The greater the strength of the irritation, the more dispersed and extensive the movements induced,—the more prolonged the irritation (irrespective of strength), the more readily are the reflex movements produced. In paraplegics, tickling more readily excites reflex movement than pinching. Superficial inflammation of membranes is accompanied by greater reflex action than deep-seated and severe inflammation, thus, in superficial inflammation, of the respiratory mucous membrane, there is great coughing; of the nasal mucous membrane, sneezing; worms in the intestines rather produce nervous disturbance than enteritis; many purgatives operate well in small doses, but cause colic and stoppage in large.

Abnormally increased sympathy may be owing to a pre-existing excited state of the primarily affected nerve, the sympathetically affected nerve or of both at the same time. The occurrence and spread of sympathies are favored by the pre-existence of excitement and increased excitability of the nerves from which the sympathy sets out; thus, in severe inflammation of the eye, spasmodic closure of the lids, great contraction of the pupil, and lachrymation, which in the natural state are excited only by strong light, are excited by moderate light. Causes which would otherwise produce slight inflammation, will, in an excited state of the nerves, produce severe inflammation. The occurrence and spread of sympathy are favoured by increased excitement, and excitability of the nerves which secondarily take part in the excitement of the primarily affected nerves, for example, a paroxysm of neuralgia, will come on from motion of the neighbouring muscles.

Henle draws the conclusion that there is no particular organic disposition to sympathies, and that the spread of sympathy depends alone on the strength and kind of the irritation, and on the degree of excitability, or, which is the same thing, the degree of excitement.

### COURSE AND EVENTS OF DISEASE.

1. COURSE.—*a. Duration. Type.* The oldest classification of diseases is that, according to their course, into *acute* and *chronic;* but though the difference be great, as respects course, between inflammation of the brain and phthisis, there are diseases which cannot well be referred to the head either of acute or chronic.

Three conditions are implied in the ideas of *acute* and *chronic.* First, the ideas refer to the absolute *duration* of the disease, *acute* is = rapid in course, *chronic* = slow in course; but in this point of view, the division has the value merely of an artificial system, as under it what is closely related may be widely separated. Secondly, the ideas acute and chronic were resolved into *febrile* and *non-febrile,* but although this holds very generally, still it is to be remembered that fever often occurs in chronic disease in its latter stages, and is sometimes absent in acute diseases. Thirdly, acute diseases were defined as diseases running through a certain length of course, and through an evident succession of stages; chronic, on the contrary, as irregular in their course, without decided progress to recovery or death. According to this view, acute is synonymous with *typical,* chronic with *atypical.* But in order to understand rightly the meaning of this distinction, and to judge of its value, it is necessary to take into consideration the foundation of type and the signification of the word.

We arrive at the type of a species, of a genus, of a family, when, on comparison of a series of individual species and genera, we recognise the accidental and peculiar characters, from the general and constant. The type of the species is, therefore, the law for the form of the individuals, and the form of the individuals is typical, or regular. But it is not the form merely which is typical, but also the development and the reactions on external irritants. When reactions are typical, diseases are so also, for disease is, according to the definition above given, nothing more than reaction against unusual irritants.

Properly there are none but typical diseases, but diseases are distinguished into *typical* and *non-typical;* diseases with regular and irregular course. On what is this distinction founded? With particular reference to their development in respect of *time*, those diseases are called typical which, besides the other specific characters by which several cases are recognised as belonging to the same species, resemble each other in their course; diseases, on the contrary, are called atypical when the course is subject to considerable deviations, without any evident specific difference to explain it. In general typical diseases are such as arise from causes operating once, and that quickly; the non-typical, on the contrary, such as are produced by causes operating gradually or constantly. Moreover, typical diseases are at the same time *pure*, whilst non-typical are in general identical with *specific, complicated, constitutional*, modified by the influence of predisposing causes. Lastly, typical diseases are those to which there belongs no other than the most general predisposition, whilst, on the contrary, most non-typical diseases presuppose a particular morbid, even hereditary predisposition.

In regard to the question how far the ideas typical and non-typical correspond to the ideas *acute* and *chronic*. It has been shown that neither the absolute duration, nor the accompanying fever, are characters according to which diseases may be naturally separated and divided into groups. It is otherwise with the type. Whether the course be regular or not, this depends, as just shown, on the most essential difference, viz. the etiological condition; it is of immediate influence on the determination of the nature of the whole morbid process, nay, even on the treatment, for it directly results that this must, in non-typical diseases, be also directed to the removal of the predisposing disease or the still operating cause. If then the words *acute* and *chronic* are to be used, as hitherto, to designate the two principal classes of diseases, which have been separated without reflection, it appears proper to impart to them the ideas *typical* and *atypical.*

It is accidental that most pure typical diseases are at the same time rapid in their course and febrile, and that most complicated or atypical diseases are at the same time slow in their course and without fever. The first are febrile, the second non-febrile, because fever symptoms, like so many other reactions which have their source in the nervous system, arise only from influences which operate suddenly and quickly.

Typical and acute cannot thus be taken as identical; for although the idea acute must comprehend more than duration, it nevertheless excludes slow diseases. In this case, acute diseases would thus comprise only a part of the typical diseases, viz. the miasmatico-contagious, besides congestions and inflammations, the course of which is always rapid and progressive, and which, when the attack is at all severe, are usually accompanied by fever. Acute is thus almost identical with inflammatory.

In the course of acute diseases to death or recovery, there may, for the most part, be recognised a succession of distinct stages, of which *five* are pretty generally admitted.

1. The stage of commencement or premonitory stage (*St. opportunitatis, St. prodromorum*, and, as regards contagious diseases, *St. latentis contagii*). This sometimes appears suddenly with marked rigors; sometimes gradually with paleness, shivering, pains all through the body, nausea and anorexia, even slight fever, symptoms which are common to many diseases, and which do not yet indicate the specific form of the coming disease.

2. The stage of progression (*St. incrementi*), from the appearance of the essential symptoms to their full development.

3. The height of the disease (*St. acmes, staseos*), called also the stage of the crisis.

4. The stage of decline (*St. decrementi*).

5. The stage of recovery (*St. reconvalescentiæ*).

*b. Periodicity—Rhythm.* A disease is rhythmical or periodical, when one or all of its symptoms alternately rise and decline or disappear, in certain more or less regular periods of time, without any external cause having occasioned the increase. When there is no rhythmical rise and fall the disease is said to be continued.

Rhythmical disease may be at the same time typical or atypical, acute or chronic.

In rhythmical disease the organic condition of it continues even during the intermission; it is thereby distinguished from *recurrent disease*, which is a new disease called forth in a case of predisposition, by a newly applied external cause.

There are only two causes to which the periodicity of disease is referable; the injurious influence either operates periodically, or the morbid symptom depends on the changed action of an organ, the healthy life of which is periodical. We have first to determine the *cause* of periodicity in the normal processes of life. Then, in order to investigate the cause of periodicity in particular diseases, it is necessary to seek out, from among the physiological processes, those which are rhythmical. For all the processes of life are not rhythmical though all are typical.

Under the head of periodicity in health, Henle considers the normal periodicity of sleeping and waking, the periodical differences in the frequency of the pulse and respiration, and in the quantity of carbonic acid and animal heat generated, the periodicity of menstruation, &c., and then observes, in regard to *periodicity in disease*:—It was seen, in considering the healthy rhythmical functions, that in the life of the nerves a daily periodicity prevails. A continued organic change must therefore exhibit daily remissions, because the reaction of the nerves increases and remits rhythmically and in daily periods. It is probable that the rhythms of several days, at least the rhythm of three to four days, is owing to the same cause, seeing that the quotidian rhythm passes into the tertian and quartan, and *vice versâ*, and seeing that it is in accordance with the laws also of normal periodicity, that the duration of the periods immediately becomes doubled, when from any cause an exacerbation or remission fails to take place. Thus hunger, desire to go to stool, sleep, menstruation, if once suppressed, do not recur immediately after the suppressing cause has ceased, but not until the next normal period. There are, however, on the other hand, reasons to suspect that there is a rhythm in the organs of the nutritive functions also, which comprises a series of days. For it is remarkable, that in quartan fever the disturbance of nutrition is so much more marked, and the affection of the viscera so much more considerable, though supposing the paroxysm be viewed as excitement, the longer duration of the intermission may indicate a slighter affection of the nervous system. If six to seven days' periods in normal nutrition be admitted, the whole duration of many diseases would embrace exactly one or several such periods. The monthly rhythm of diseases is readily explained by the periodicity of the sexual functions in the female, and of nutrition in both sexes. Even the annual course of certain morbid symptoms may be referred to a yearly rhythm in the growth of the body.

Henle would thus, speaking generally, refer morbid symptoms with daily rhythm to the nervous system; with monthly rhythm to the female sexual organs, or to the organs of nutrition; with annual rhythm to the organs of nutrition.

2. OF THE EVENTS OF DISEASE. Disease ends either in recovery or in death, or it passes into a new disease.

*Recovery.* When a disease ends in recovery, without the interference of art, the recovery is said to take place *spontaneously*, or by the *efforts of nature;* but, properly speaking, recovery is *spontaneous* only when it is altogether independent of new influences of any kind, accidental as well as intentional; in other cases recovery is *by art,* or is *accidental.*

Spontaneous recovery may take place in two ways, viz.—1, in certain cases by the simple expulsion, or cessation of the cause; 2, in other cases in which the morbid process has continued, notwithstanding the removal or cessation of the cause, by the supervention of some action or event to which the morbid process itself has at last led, such as hemorrhage, the bursting of an abscess, &c. In all this there is no ground for the admission of a *vis medicatrix Naturæ.* Even if the process from disease to recovery were less clear than it is, an explanation which, notwithstanding its modern metamorphoses, still betrays its mythical origin, should be rejected.

The formative power with which the different structures are endowed is not roused by irritation for the protection of the body, but is rather turned from its object, and that to the injury of the body. *After* the irritation the conditions are re-established, under which it operates normally. Every *reaction* is a morbid symptom, it is the rest and *restitution* which succeed the irritation which are curative.

In addition to this power, which operates in the preservation of the individual during the disease, inasmuch as it operates generally for the preservation of life, sympathy and antagonism are often the means by which prejudicial influences are rendered innoxious and diseases cured. The injury, by the accidents it occasions, often brings about the cure. Irritation of the mucous membrane of the stomach excites vomiting, irritation of the glottis, coughing, and by means of these sympathetical movements matters are sometimes expelled, the continuance of which in the body would have been injurious. But that sympathy is eternal, this use merely accidental! Every poison does not occasion vomiting, nor every noxious gas coughing. Coughing may arise from superficial inflammation of the trachea before there is anything to expectorate, and vomiting from injection of tartar emetic into the veins when evacuation of the stomach is quite useless.

*Crisis.* This name has been given to the sudden disappearance of the symptoms of a disease after some marked evacuation. The idea of crisis is a remnant of the mythical beginnings of medicine. According to the ancient views, crisis is the expulsion, by the curative powers of nature, of a *materia peccans.* The symptoms of excitement of pulse, increased heat, delirium, spasm, &c., were viewed as manifestations of the action by which the *materia peccans* was elaborated and its evacuation brought about, and therefore called critical exacerbation, or *molimen criticum.*

Testing the question of crisis empirically, Henle shows, from the statistical observations of late years, how small the number of cases is in which what could be called a crisis occurred in comparison with the number of those in which there was nothing of the kind.

*Transition into another disease.* The disease which passes into another undergoes a change of form or place.

In regard to change of form (*Metaschematismus*) many different distinctions have been made, as, for example, the conversion of acute into chronic disease, of one general disease into another, of one local disease into another, &c.; but all that has been or can be said on the subject is worthless, if regard be not at the same time had to the cause of the disease. If a particular combination of symptoms is changed, in consequence of new causes, into another, it is evidently a figure of speech merely to call this the transition of one disease into another. It is really a new disease in the place of another. But if the second disease is developed out of the first, without new influences, as, for example, when congestion becomes inflammation, both are merely stages of one and the same process, and it is improper to separate them from each other under specific designations.

The idea of change of place of disease or *metastasis* had its origin, like that of crisis, in the mythical pathology. The materia peccans, supposed to be wandering about in the body in disease, was, according to that pathology, in crisis excreted by an organ not itself diseased, whilst in metastasis it was supposed to throw itself on an organ and occasion disease in it, a disease, however, sometimes curative, and then called *critical.* The transference of a disease from within outwards, with benefit to the patient, was thus also called crisis; and transference of a disease from without inwards, with injury to the patient, metastasis, in the limited acceptation.

Modern humoral pathology has sought, in accordance with the enlargement of chemical and physiological knowledge, to determine more accurately the nature of the supposed materia peccans, and the way in which it wanders. In regard to the first point, it has scarcely done more than institute hypotheses; and as to the second, it has agreed that the blood-vessels and absorbents convey the morbid matter, taking it up from one place and depositing it in another. When a general disease passes into a local, it supposes that the blood deposits in a particular organ the matters which occasioned the impurity of it, on which the general disease depended. When, on the contrary, a local disease passes into a general, the blood takes up the morbid matter from the particular part affected.

According to this modern view, a disease, in order to be recognized as metastatic, must be the consequence of the cessation or non-appearance of another, and that what in the case of the one disease is repelled, and breaks out in the other, is a constituent of the blood. In the application of that idea of metastasis, a double error has been committed :—1, the causal connexion has been, often on too light grounds, presupposed, and any *second* disease, without farther inquiry, taken for a *secondary* one ; 2, on the strength of the principle of explanation adopted, a number of hypothetical morbid matters, has been admitted, and the multifarious means by which the anormal action might be transferred from one organ to the other overlooked.

It is thus necessary to strike out from among the metastases the cases in which the supposed causal connexion does not exist, and in which the disease, erroneously put down as metastatic, is not owing to the first having ended. Having excluded the pseudo-metastases, Henle adduces the different modes in which true metastasis comes about, i. e. the different modes in which the arrestment of morbid processes gives occasion to the origin of morbid vital manifestations in other places ; but he observes in regard to them that there is not one which perfectly corresponds to the definition of metastasis as given by the humoral pathologists. The common character is indeed transference of the morbid process, but in no case does this take place by the vessels taking up and transporting an already existing morbid deposit from one place to another. He does not indeed call in question the possibility of such a process, but says that its actual occurrence must be only under rare conditions, and suggests, as an example, certain of the so-called purulent metastases ; those cases, viz. in which the sudden collapse of unopened abscesses is followed by the appearance of a sediment in the urine, purulent effusion into the serous sacs, or copious expectoration of matter from the lungs. In such a case, however, he remarks, that it could be the plasma only of the pus which is absorbed and again deposited ; the corpuscles must remain where they were, and the corpuscles in the newly deposited pus must be of new formation.

*Death.* Death should be called natural, necessary or normal only when it occurs after the completion of the typical period of life. Death under any other circumstances should be called unnatural, accidental, or abnormal.

Death is cessation of the nutritive interchange. The sign of it is definitive stoppage of the function ; if the function of an organ is striking, its death is soon recognised ; in the opposite case the death of a part may remain for some time undetected. When the hairs in typhus fever die, the circumstance is not recognised ; until they begin to fall out during convalescence; death of the brain, on the contrary, is instantly recognised.

All possible causes of accidental death, as well of the whole organism, as of individual organs, may be grouped under the two following heads.

1. Want of vital excitants, viz. oxygen and heat, which are supplied to the individual organs by the blood.

2. Alteration of the organic substance by physico-chemical influences, by which it becomes incapable of the typical reception of vital excitants.

Death may be like disease, general or local. Death is general when the destructive influence strikes all parts at the same time, as in the case of electricity and prussic acid, which at once destroy irritability in all the tissues. Local death is the result of a locally acting influence, or a local disease. Local death, even the inactivity of organs, occasioned by temporary conditions, by the action of which the vital excitants are supplied to the others, necessarily induces general death. Such organs are the heart, lungs and medulla oblongata. These organs stand in a peculiar reciprocal relation: each sends to the other and receives from it the conditions of its vital manifestations. The innervation of the respiratory muscles depends on the medulla oblongata, the energy of the medulla oblongata on the decarbonization of the blood, in effecting which circulation and respiration must combine. Thus the loss of any one of the three links of the chain is fatal.

The outline which has now been given of Professor Henle's work will, we believe, afford the reader an idea of its spirit and scope, and justify the opinion as to its general excellence, expressed at the commencement of this article. We have thought it unnecessary to enter into any criticism of details.

---

### Art. II.

*Medico-Chirurgical Transactions.* Vol. XXIX.—*London,* 1846. 8vo, pp. 352. Eleven Plates.

